# Climate Change Adaptation: Prehospital Data Facilitate the Detection of Acute Heat Illness in India

**DOI:** 10.5811/westjem.2020.11.48209

**Published:** 2021-03-24

**Authors:** Nikhil Ranadive, Jayraj Desai, LM Sathish, Kim Knowlton, Priya Dutta, Parthasarathi Ganguly, Abhiyant Tiwari, Anjali Jaiswal, Tejas Shah, Bhavin Solanki, Dileep Mavalankar, Jeremy J. Hess

**Affiliations:** * University of California, San Francisco-Fresno, Department of Emergency Medicine, Fresno, California; † University of Washington, Center for Health and the Global Environment, Seattle, Washington; ‡ GVK-Emergency Management and Research Institute, Department of Emergency Medicine Learning & Care (Gujarat), Gujurat, India; § Indian Institute of Public Health, Gandhinagar, Gujarat, India; ¶ Natural Resources Defense Council, New York City, New York; || Ahmedabad Municipal Corporation, Gujarat, India; # University of Washington, Department of Emergency Medicine, Seattle, Washington; ** University of Washington, Department of Environmental and Occupational Health Sciences, Seattle, Washington; †† University of Washington, Department of Global Health, Seattle, Washington

## Abstract

**Introduction:**

Extreme heat is a significant cause of morbidity and mortality, and the incidence of acute heat illness (AHI) will likely increase secondary to anthropogenic climate change. Prompt diagnosis and treatment of AHI are critical; however, relevant diagnostic and surveillance tools have received little attention. In this exploratory cross-sectional and diagnostic accuracy study, we evaluated three tools for use in the prehospital setting: 1) case definitions; 2) portable loggers to measure on-scene heat exposure; and 3) prevalence data for potential AHI risk factors.

**Methods:**

We enrolled 480 patients who presented to emergency medical services with chief complaints consistent with AHI in Ahmedabad, India, from April–June 2016 in a cross-sectional study. We evaluated AHI case definition test characteristics in reference to trained prehospital provider impressions, compared on-scene heat index measured by portable loggers to weather station measurements, and identified AHI behavioral and environmental risk factors using logistic regression.

**Results:**

The case definition for heat exhaustion was 23.8% (12.1–39.5%) sensitive and 93.6% (90.9–95.7%) specific. The positive and negative predictive values were 33.5% (20.8–49.0%) and 90.1% (88.5–91.5%), respectively. Mean scene heat index was 6.7°C higher than the mean station heat index (P < 0.001), and station data systematically underestimated heat exposure, particularly for AHI cases. Heat exhaustion cases were associated with on-scene heat index ≥ 49°C (odds ratio [OR] 2.66 [1.13–6.25], P = 0.025) and a history of recent exertion (OR 3.66 [1.30–10.29], P = 0.014), while on-scene air conditioning was protective (OR 0.29 [0.10–0.85], P = 0.024).

**Conclusion:**

Systematic collection of prehospital data including recent activity history and presence of air conditioning can facilitate early AHI detection, timely intervention, and surveillance. Scene temperature data can be reliably collected and improve heat exposure and AHI risk assessment. Such data may be important elements of surveillance, clinical practice, and climate change adaptation.

## INTRODUCTION

Extreme heat is a significant cause of morbidity and mortality globally.^1^–^8^ Heat poses a threat to human health both directly, causing acute heat illness (AHI) such as heat exhaustion and heat stroke, and indirectly, by exerting stress on physiological systems and exacerbating chronic diseases.^9^–^11^ Acute heat illness results from both exogenous and endogenous heat exposure. Exogenous exposure to extreme ambient temperature will likely continue to increase due to the increasing frequency of extreme heat events and anthropogenic climate change.^12^,^13^ However, endogenous exposure from exertion is also a significant and increasingly prevalent risk factor.^14^

Regardless of the exposure pathway, prompt AHI diagnosis and treatment significantly improve clinical outcomes.^15^ Acute heat illness is a clinical diagnosis facilitated by a high index of suspicion as well as historical and other data that can help determine exposure to endogenous and exogenous heat sources. This is particularly important in the prehospital setting, where diagnostic uncertainty is high, access to adjunct laboratory and other tests is limited, and critically important historical data can be gathered. There is an unmet need to design, test, and evaluate tools to facilitate the early recognition and treatment of AHI in the prehospital setting and to facilitate public health surveillance. This deficit is particularly relevant in India, China, and other low- and middle-income countries (LMIC), which are disproportionately impacted by climate change and where extreme heat poses substantial risk.^16^–^19^

In this study we evaluated prehospital AHI diagnostic tools in Ahmedabad, Gujarat, India, a city experiencing significant warming including a heat wave in 2010 that led to more than 1344 deaths – a 43.1% increase over the baseline mortality rate.^9^ We conducted an exploratory evaluation of three tools with the potential to facilitate early recognition of AHI and more accurate public health surveillance: 1) heat exhaustion and heat stroke case definitions for identifying likely AHI cases; 2) portable digital temperature and humidity data loggers that can be used to measure real-time on-scene temperature at the time of first responder arrival; and 3) prevalence data for AHI risk factors contained in prehospital provider history and physicals (H&P) that can be used to facilitate early diagnosis. We evaluated the potential utility of these tools individually and in combination for improving diagnostic accuracy for AHI in the prehospital setting.

## METHODS

### Study Overview

This study, which includes a retrospective and exploratory diagnostic accuracy evaluation and a cross-sectional analysis, was conducted between April–June 2016 in Ahmedabad, India.

### Study Setting and Collaboration

Ahmedabad is the sixth largest city in India. It has a population of 7.2 million people and is among the fastest growing cities in the country. It is also one of India’s hottest cities, with summer maximum daily temperatures (T_max_) averaging 45°C from March–May.^9^,^20^–^22^ Like other industrializing cities in LMICs, several populations in Ahmedabad have a high risk for heat illness, including residents of slums and densely populated areas, individuals with limited access to water and air conditioning, and laborers in a range of settings.^23^–^26^

**Population Health Research Capsule**

What do we already know about this issue?

*Climate change is increasing acute heat illness (AHI) prevalence. Prompt diagnosis is key. Exposure history is important, but diagnostic tools are limited.*

What was the research question?

*Can prehospital data elements facilitate the early identification of AHI?*

What was the major finding of the study?

*Prehospital data such as activity history and scene temperature measurement can improve rapid AHI diagnosis.*

How does this improve population health?

*Such data may be important elements for timely surveillance and treatment as the disease burden attributable to extreme heat rises in the setting of climate change.*

This study was conducted in partnership with the Ahmedabad Heat and Climate Study Group and the GVK-Emergency Management and Research Institute (EMRI). GVK-EMRI is the largest emergency medical services (EMS) provider in India and has an active research program. The Ahmedabad Heat and Climate Study Group is comprised of the Ahmedabad Municipal Corporation, the Public Health Foundation of India, the Indian Institute of Public Health-Gandhinagar, the Natural Resources Defense Council, and an international coalition of academic partners including researchers from the University of Washington and the Icahn School of Medicine at Mount Sinai, New York.^27^,^28^ Since 2009 the group has developed evidence-based heat preparedness through a heat action plan that combines forecasting of extreme heat, threshold-based early warnings, and capacity building of local health professionals, including training of GVK-EMRI emergency medical technicians (EMT) and their online medical support.^27^

### Study Sample and Data Collection

**Figure 1:**
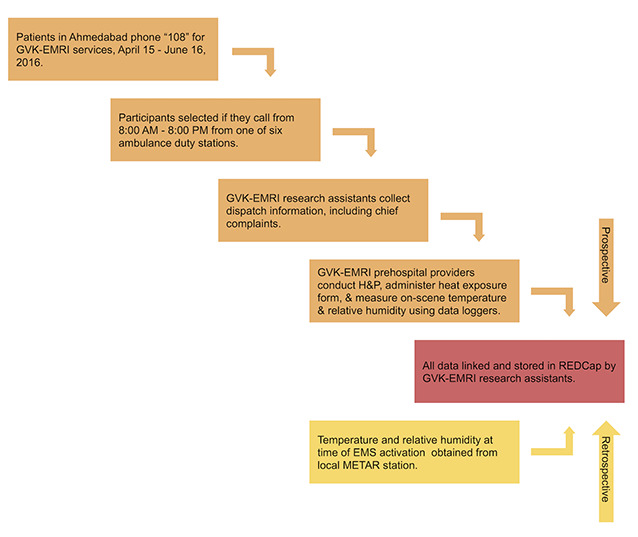
Study diagram, including participant enrollment, data collection, and data storage. *GVK-EMRI*, GVK- GVK-Emergency Management and Research Institute; *H&P*, history and physical; *EMS*, emergency medical services; *METAR*, Meteorological Terminal Aviation Routine weather report.

A flow chart of study enrollment and data collection is shown in Figure 1. We used a convenience sample of patients who were included if they presented to the GVK EMRI-operated “108” ambulance service from 8 am – 8 pmwith an eligible chief complaint during the study period and were served by one of six ambulance duty stations with the highest historical call volume for AHI. A list of eligible chief complaints consistent with AHI was developed a priori by the study team and included chest pain, abdominal pain, shortness of breath, intoxication, hypertension, hyper- or hypoglycemia, syncope, dysrhythmia, headache, mental health concerns, seizures, stroke, altered mental status, fever, and nausea and vomiting. All trauma activations were excluded from the study. Patients were enrolled from April 15–June 15, 2016. We collected exposure and outcome data using a number of retrospectively and prospectively accessed data streams. Clinical and demographic data were collected and entered into an online database by trained research assistants in the GVK-EMRI dispatch center. Prospectively, GVK-EMRI research assistants collected dispatch information; demographics, including educational attainment, a proxy for health literacy; and chief complaint data when patients phoned for EMS. GVK-EMRI prehospital providers then collected history and physical data using standard H&P methods as well as through administration of standardized data collection instruments, which included questions about possible heat exposures and heavy physical activity prior to the event (Methods Supplement); these data were then entered into a password-protected and Health Insurance Portability and Accountability Act-compliant database (REDCap) by the research assistants. Prehospital provider impressions regarding the need for emergent cooling and overall clinical impressions were included in the questionnaire.

We collected environmental data from two sources: portable data loggers and the Ahmedabad airport weather station. On-scene temperature and relative humidity (i.e., at the location of patient pick-up) were measured using portable data loggers that were attached to the prehospital providers’ stretchers and subsequently transported onto the scene. The Lascar EL-USB-2-LCD USB Humidity Data Loggers were developed by Lascar Electronics (Whiteparish, England), a global company that designs custom-made data logging tools. Using an internal sensor, the loggers equilibrated with ambient temperature and humidity in approximately one minute. Temperature and humidity were automatically logged every 30 seconds and averaged. To allow for equilibration, only temperature and humidity data timestamped two minutes after scene arrival were included for analysis. Hourly weather data (temperature and relative humidity) were also accessed retrospectively from Ahmedabad’s Meteorological Terminal Aviation Routine weather report (METAR) station at the Sardar Vallabhbhai Patel International Airport, which is geographically surrounded by the city of Ahmedabad. All data streams were then linked and stored in REDCap, including EMS activation times, which were linked to the nearest corresponding METAR station data points.

### Prehospital Provider Approach to Recognizing and Managing Heat Illness

The prehospital providers in this study were EMTs with basic certification who underwent a rigorous selection and training process conducted in collaboration with the Department of Emergency Medicine at Stanford University. This involved 42 days of training, Basic Life Support and International Trauma Life Support certification, and refresher training every six months. All GVK-EMRI prehospital providers have received specific training for environmental emergencies including heat exhaustion and heat stroke, use protocols for identifying and treating heat illness developed by Stanford Emergency Medicine, and have a high index of suspicion for AHI and the need for implementing cooling interventions.

The protocol for AHI outlines signs and symptoms for heat illness across the spectrum of heat cramps, heat exhaustion, and heat stroke (including cramps, headache, fatigue, nausea and vomiting, and, for heat stroke, anhidrosis, altered mental status, and a temperature over 40°C); and management priorities including exposure, rapid cooling, intravenous hydration, and assessment and management of hypoglycemia and seizures, and transport. Indications for contacting online medical control include temperature over 40°C or altered mental status. Given the intense heat in Ahmedabad during the summer season, prehospital providers are familiar with AHI presentations and experienced in their management. Lastly, prehospital providers received additional assistance from qualified online medical control physicians in real time, and were routinely subject to quality control and medical audits.

### Data Analysis

Data cleaning and statistical analyses were conducted using Stata/MP 15.1 (StataCorp, College Station, TX), Tableau Desktop 2019, and Tableau Prep Builder 2019 (Tableau Software, Seattle, WA). Main outcome measures included 1) test characteristics of AHI case definitions; 2) exposure assessment comparing on-scene and weather station heat index means and correlations between the two measures for the entire sample and AHI cases; and 3) odds ratios (OR) for AHI risk factors. All analyses used prehospital provider impressions to identify AHI cases.

#### Evaluating the diagnostic accuracy of AHI case definitions

We conducted a retrospective and exploratory diagnostic accuracy evaluation of case definitions for heat exhaustion and heat stroke (developed by the study team) using prehospital provider clinical impressions and the initiation of cooling in the prehospital setting as a reference standard. The heat exhaustion case definition included feeling hot with a complaint of any of the following: nausea, vomiting, dizziness, weakness, diarrhea, fainting, muscle cramps, hot and dry skin, hot and diaphoretic skin, or headache. The heat stroke case definition included a core temperature of at least 38.5°C, with altered mental status (Glasgow Coma Scale less than or equal to 14, disorientation, seizures, or loss of consciousness). All temperatures obtained in the axilla were adjusted upward by 1°C to more accurately reflect internal (i.e., rectal) temperatures.^29^

Regarding selection bias and uncertainty, index text results (i.e., the case definitions) were not available to prehospital providers in the field; however, the study authors were not blinded to prehospital provider impressions while retroactively developing the case definitions. While we did not conduct sample size calculations, we anticipated a sample size of 300–600 participants based off of historical EMS call activity in the area during the summer months. We also conducted a sensitivity analysis using multiple prevalence estimates to calculate positive and negative predictive values. Prevalence estimates were obtained from a previously conducted prevalence study of self-reported heat-related symptoms (20.1%) and heat-related illness (11.9%) among slum dwellers in Ahmedabad.^25^

#### Evaluating portable data loggers

Data from portable data loggers were tested to compare the utility of on-scene vs city-level (i.e., METAR) heat exposure data in assessing AHI risk. In the past, meteorological data obtained from airport stations have been routinely used to characterize exposure in heat-health studies; however, these may not adequately reflect city-center conditions.^30^ To more accurately assess heat exposure we calculated the heat index, which incorporates both temperature and relative humidity, using the US National Weather Service heat index algorithm for both logger and station data.^31^ We compared the heat index as measured by the data loggers with temperature measurements from the airport station and evaluated the correlation using Spearman’s rho. Unpaired, one-sided t-tests were used to compare the difference in the mean heat index between AHI cases and non-cases (hypothesized to be higher among cases), as diagnosed by prehospital providers. Subgroup analysis was conducted for participants with a reported history of recently experienced exertion, which may confound heat-health relationships. We also conducted a paired t-test to compare the mean logger heat index to the mean station heat index, specifically among AHI cases (as defined by prehospital providers).

#### Assessing risk factors and heat-health relationships

We conducted a number of logistic regression models using each case definition as a dichotomous outcome variable (i.e., heat exhaustion and heat stroke cases and non-cases) to better characterize heat-health relationships, ccount for confounding variables, and identify risk factors for possible use in a clinical decision-making tool. Models were evaluated and selected using the chi-square goodness-of-fit test and Stata’s linktest function, which assesses specification error. Independent variables in each model included heat exposure data such as logger and station heat indices, station visibility (a surrogate for air quality, as air quality data such as daily particulate matter and ozone levels was not available), and station wind speed, and variables to distinguish between exertional and non-exertional heat illness (i.e., a report of recently experienced heavy labor).^32^ Given the clinical utility of thresholds, logger and station heat indices were included as dichotomous covariates with temperature thresholds ≥ or < 49°C (consistent with previously described heat-wave temperature thresholds in South Asia), rather than as a continuous variable.^33^

### Ethics

Ethics approval was obtained from the Indian Council of Medical Research (TRC/IEC No. 14/2015), the University of Washington (#51167), and the Indian Institute of Public Health, Gandhinagar. A waiver of informed consent was obtained to access medical records, and all patient information was de-identified prior to analysis.

### Disclosures

All authors completed the ICMJE uniform disclosure form at http://www.icmje.org/coi_disclosure.pdf and declare the following: no support from any organization for the submitted work; no financial relationships with any organizations that might have an interest in the submitted work in the previous three years; and no other relationships or activities that could appear to have influenced the submitted work.

### Funding

This work was funded by the National Institutes of Health (grant number 5R21TW009535-02). The funding source had no involvement in study design, data collection or analysis, manuscript writing, or the decision to submit this manuscript for publication.

## RESULTS

### Demographic, Environmental, and Clinical Characteristics

**Figure 2:**
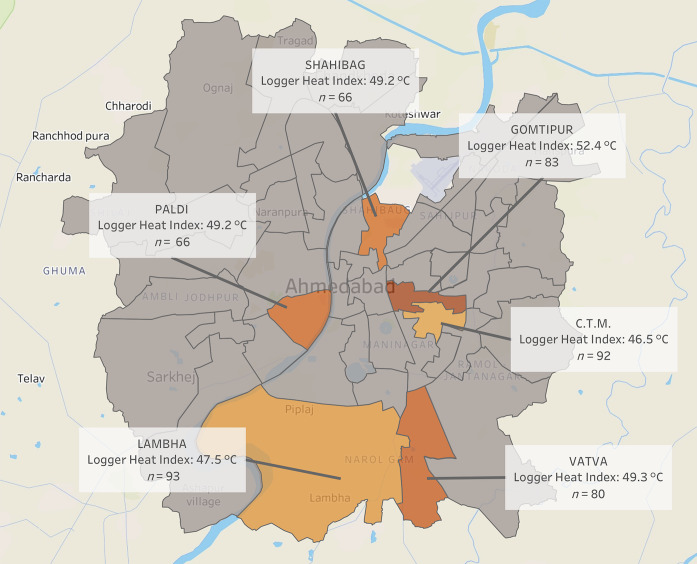
Mean logger heat index and emergency medical services call volume (n) for each of the six eligible ambulance duty stations in Ahmedabad, India. *C*, Celsius.

Study sample characteristics and environmental exposure data are described in Table 1 (see Figures S1 and S2 for participant flow diagrams). A total of 480 participants were enrolled into the study, with 49.8% males and 50.2% females. The median age was 41.5 years with an age range of 1–95 years. At total of 349 participants (72.71%) reported an educational attainment of primary school or less, and 21.5% of participants reported a history of chronic disease. Due to technological malfunctioning of the data loggers, logger temperature and relative humidity were collected for 415 and 379 of the 480 participants, respectively, and missing values were dropped from the analysis. The median logger and station temperatures for all calls were 43.0°C and 40.6°C, respectively. The humidity-adjusted logger and station heat indices were 50.2°C and 44.2°C, respectively. The majority of individuals in the study (83.96%) reported indoor (vs outdoor) occupations. Twenty-six individuals (5.4%) reported a history of recently experienced exertion prior to phoning for EMS. Mean logger heat indices stratified by ambulance duty call station ranged from 46.5–52.4°C, and are displayed in a map in Figure 2. Objective signs and physical exam findings are described in Table 1. Fifty-nine participants (12.3%) had hot and dry skin according to prehospital providers. The majority of participant temperatures were taken in the axilla; when adjusting for core temperatures, over 100 patients (25.4%) had core temperatures greater than 38.5°C, and 47 patients (9.8%) had core temperatures over 40°C. Over 30% of participants endorsed weakness, nausea, and vomiting, and 28.6% of participants had a chief complaint of syncope.

### Evaluation of Case Definitions

The sensitivity, specificity, positive predictive values (PPV), and negative predictive values (NPV) of the heat exhaustion case definitions using prehospital provider impressions as the reference standard are shown in Table 2. The heat exhaustion case definition had a sensitivity of 23.8 (12.1–39.5), while the heat stroke case definition sensitivity was 100% (29.2–100.0%). The heat stroke test characteristics had wide confidence intervals due to a small number of heat stroke cases (*n* = 3).

### Evaluation of Data Loggers

**Figure 3:**
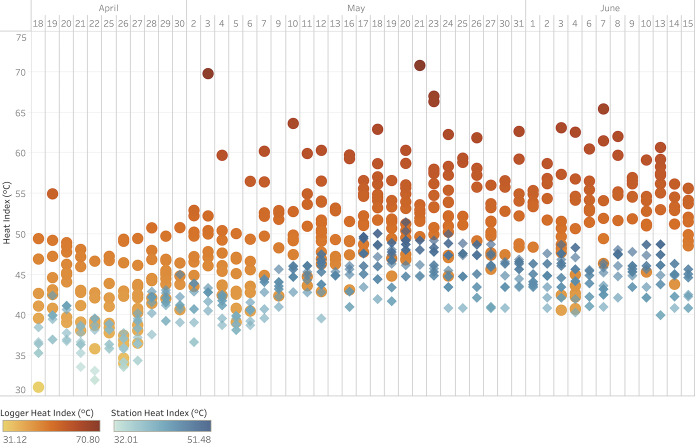
Daily logger (red circles) and station (blue rhombi) heat indices for all study participants. *C*, Celsius.

A side-by-side comparison of daily logger and station heat indices is shown in Figure 3, which demonstrates systematically warmer logger heat indices throughout the study period and increased variability in logger heat indices at station heat indices ≥ 45°C.

**Figure 4:**
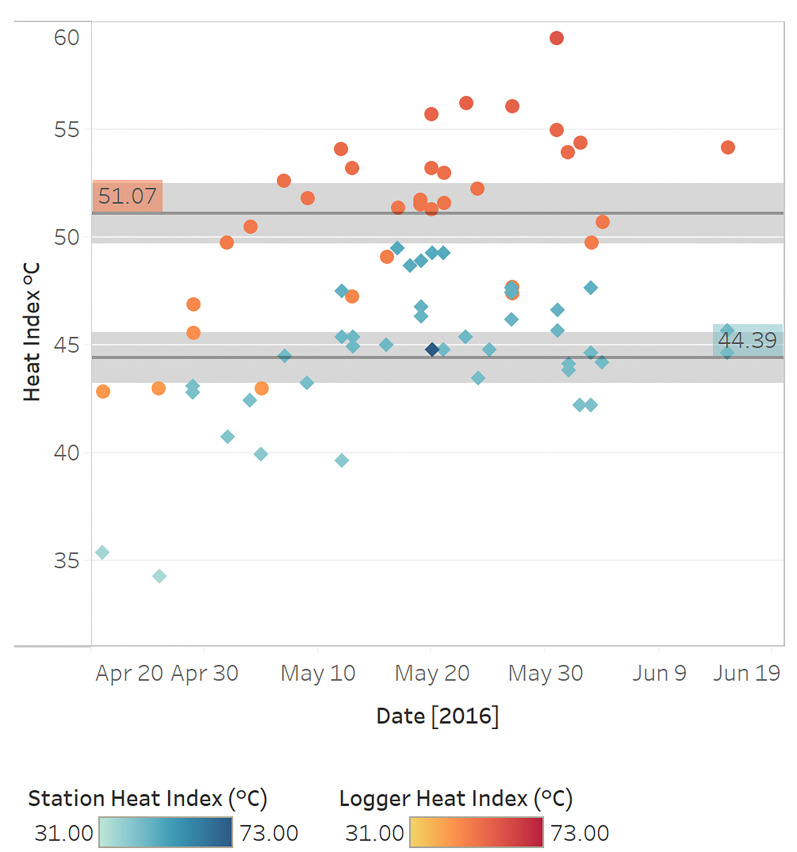
Mean heat indices (HI) with 95% confidence intervals as measured by loggers (red circles, upper band) and the METAR* station (blue rhombi, lower band) among individuals with heat exhaustion according to pre-hospital providers. *C*, Celsious; *METAR*, Meteorological Terminal Aviation Routine weather report.

We evaluated heat index differences between heat exhaustion cases and non-cases (as determined by prehospital providers) stratified by measurement modality using one-sided t tests (α = 0.05) (Table S1). The mean on-scene heat index among heat exhaustion cases was 1.2°C higher than for non-cases (*P* = 0.162). When restricting this analysis to patients reporting a history of recently experienced exertion, the mean on-scene heat index for heat exhaustion cases was 0.9°C higher than for non-cases (*P* = 0.394). The mean airport station heat index among heat exhaustion cases was 1.1°C higher than for non-cases (*P* = 0.037). The mean station heat index for heat exhaustion cases remained 0.9°C higher than for non-cases when restricting the analysis to patients with a history of recently experienced exertion (*P* = 0.327). Figure 4 displays the mean logger and station heat indices for heat exhaustion cases. Using a paired t-test (α = 0.05), the mean logger heat index was 6.7°C (*P* < 0.001) higher than the mean station heat index among heat exhaustion cases, as defined by prehospital providers.

### Characterization of Heat-health Relationships

A multivariate logistic regression analysis of risk factors associated with developing heat exhaustion (as determined by prehospital provider impressions) is shown in Table 3. The OR of heat exhaustion among participants with a logger heat index ≥ 49°C was 2.66 times greater than among individuals with a logger heat index < 49°C (*P* = 0.025). The OR for a station heat index ≥ 49°C vs < 49°C was 2.11, but this relationship was not statistically significant (*P* = 0.280). Heat exhaustion cases were also negatively associated with high station visibility (OR 0.69, *P* = 0.034) and access to on-scene air conditioning (OR 0.29, *P* = 0.024), and positively associated with a history of recent exertion (OR 3.66, *P* = 0.014). No statistically significant associations were found between demographic characteristics, clinical characteristics, and patient pick-up location, and these covariates were dropped from the model. We did not conduct logistic regression analysis of risk factors associated with developing heat stroke due to high collinearity and a small sample size.

## DISCUSSION

We found a significant disparity between paired logger (on-scene) and station (METAR) heat indices, with scene temperatures being systematically warmer. We observed larger differences for heat stroke cases than for heat exhaustion cases and smaller differences among AHI cases with a history of recent heavy exertion. We also observed spatial variability in logger heat indices at the level of ambulance duty stations. Taken together, this suggests that station data may not adequately capture microclimate conditions and on-scene heat exposures, and that data loggers provide useful information for exposure assessment in evaluating possible AHI cases, particularly when there is no history of heavy exertion. Both on-scene and station HI are higher for heat exhaustion cases than non-cases, although this relationship was not always statistically significant.

The observed trend is reinforced by the finding that heat exhaustion cases were significantly and positively associated with a logger heat index ≥ 49°C. Altogether we conclude that scene temperature and relative humidity may have utility as environmental tests for AHI in the prehospital setting. Prior studies have identified significant differences between monitoring station and microclimate data due to on-scene variability in wind speed and direction, solar radiation, and humidity, among other factors.^34^,^35^ A number of tools have been developed to measure on-scene heat risk, including personal temperature loggers and wet bulbs that measure radiant heat, ambient temperature, wind, and humidity.^36^–^39^ However, to our knowledge these have only been used to measure workplace risk and have not been evaluated in the context of prehospital medicine.

In the limited case series presented here, a history of exertion seems to have substantially lowered the temperature threshold for developing AHI. We also found that access to air conditioning was negatively associated with a diagnosis of heat exhaustion. Thus, while scene temperature is important, it has to be interpreted as part of total heat load, ie, including both exogenous and endogenous sources: if the patient has a history of heavy exertion or lives or works in an environment without an air conditioner, a lower scene temperature and heat index may be consistent with AHI. Prehospital providers should also specifically assess for these risk factors when obtaining patient histories, both on hot days and in areas with high AHI prevalence.

Heat exhaustion cases were also significantly associated with decreasing station visibility (a surrogate for air quality), which may support prior evidence that poor air quality modifies heat-related morbidity and mortality.^32^,^40^ While there is conflicting evidence in this area, authors of the largest study to date to assess effect modification in heat-mortality relationships found that heat-related mortality was significantly and positively associated with increased particulate matter (PM2.5).^41^ Authors of a recent meta-analysis of 21 studies found synergistic effects between high temperature, poor air quality, and non-accidental and cardiovascular mortality.^42^ Taken together, our results add to findings by Tran and colleagues, who identified a number of AHI risk factors including old age, working in the sun, and having a pre-existing chronic or infectious medical condition.^25^

Our case definition for heat exhaustion had a low sensitivity of 23.8% and positive predictive value (PPV) of 26.3%, assuming a background prevalence of 11.9%.^25^ While this performance was somewhat disappointing, it was not entirely unexpected given the low sensitivity of similar heat exhaustion case definitions in the syndromic surveillance literature. For instance, Berry and colleagues found that an AHI case definition in New Jersey based on chief complaint data (incorporating terms such as syncope, dizziness, weakness, and headache) was 16% sensitive with a PPV of 40% when using discharge *International Classification of Diseases, 9^th^ Revision* diagnosis codes as a reference standard.^43^ Similarly, the current heat syndrome case definition used for syndromic surveillance in North Carolina had a sensitivity of 16%.^44^ Given the small sample size of participants with heat stroke (*n* = 3), we were not able to make significant conclusions regarding diagnostic accuracy with these data.

## LIMITATIONS

Our study had several limitations. First, we relied on convenience sampling and a relatively small sample size. This likely underestimated the variability in on-scene temperature and the AHI predictive value of risk factors in the logistic regression analysis. Second, we relied on a passive data collection process and were not able to adequately capture and troubleshoot technological malfunctioning of the data loggers, which resulted in missing on-scene temperature and relative humidity data. Third, while our adjustment of participant temperatures obtained in the axilla were intended to better reflect internal temperatures, this correction was based on a systematic review that only included afebrile participants. Fourth, we were not able to obtain physician-confirmed diagnoses and relied on prehospital provider impressions for our reference standards. However, prehospital providers in this area have been well trained in diagnosis and management of AHI following our prior efforts to develop a heat action plan in Ahmedabad.^27^ Last, there may be an element of selection bias: prehospital providers were not blinded to the loggers or heat exposure forms when diagnosing patients with AHI, and they may have been influenced by their perception of heat at the location of patient pick-up.

## CONCLUSION

Despite these limitations, the findings from our study clearly suggest that adding additional data to prehospital evaluations for AHI can improve diagnostic accuracy, even in a setting with an ambulance service that is highly attuned to AHI. In particular, scene temperature, a history of exertion prior to illness onset, and presence of air conditioning are valuable data points. Collecting data on scene temperature is feasible and improves exposure estimation, particularly for patients with AHI. Having a standing strategy for collecting additional data regarding activity and scene environment is likely important for early AHI detection. These practice modifications can facilitate adaptation to climate change, which is increasing the frequency and severity of extreme heat events. Our findings may have particular relevance to other cities in LMICs with centrally-administered EMS systems and environmental conditions similar to Ahmedabad’s. Further studies that evaluate the use of prehospital environmental, demographic, and clinical data for the early detection of AHI are warranted.

## Supplementary Information













## Figures and Tables

**Table 1:** Study sample (n = 480) demographic, environmental, and clinical characteristics.

	n (%)
Demographic characteristics	
Gender	
Male	239 (49.8)
Female	241 (50.2)
Age (years)	
< 1	4 (0.8)
1 – 5	15 (3.1)
6 – 17	30 (6.3)
18 – 44	202 (42.1)
45 – 64	123 (25.6)
≥ 65	106 (22.1)
Highest level of education	
None/less than grade 5	237 (49.4)
Primary (up to grade 5)	112 (23.3)
Secondary (up to grade 10)	66 (13.8)
High (up to grade 12)	34 (7.1)
Bachelor’s degree or above	18 (3.8)
Missing	13 (2.7)
Prior medical history	
Alcoholism	2 (0.4)
Cardiovascular disease	8 (1.7)
Diabetes	38 (7.9)
Hypertension	66 (13.8)
Liver disease	1 (0.2)
Renal disease	9 (1.9)
Patient pickup location	
Residence	439 (91.5)
Indoor public space	4 (0.8)
Outdoor public space	14 (2.9)
Worksite	17 (3.5)
School or college	3 (0.6)
Other	3 (0.6)
Clinical characteristics	
Dermatological signs	
Skin hot, diaphoretic	21 (4.3)
Skin hot, dry	59 (12.3)
Neurological signs	
GCS ≤ 14	48 (10.0)
GCS ≤ 13	37 (7.7)
Body temperature	
≥ 38.5°C	112 (23.3)
≥ 40.0°C	47 (9.8)
Clinical characteristics	
Temperature measurement location	
Oral	81 (16.9)
Axillary	391 (81.5)
Rectal	1 (0.2)
Not measured	7 (1.5)
	Median [Interquartile range]
	
On-scene meteorological data	
On-scene temperature (°C)	43.0 [40.0 – 45.7]
On-scene relative humidity (%)	29.5 [23.5 – 38.0]
On-scene heat index (°C)	50.2 [45.6 – 54.2]
Station meteorological data	
Station temperature (°C)	40.6 [38.1 – 42.1]
Station relative humidity (%)	29.8 [20.8 – 36.1]
Station heat index (°C)	44.2 [41.2 – 46.2]

*GCS*, Glosgow Coma Scale; *C*, Celsius.

**Table 2:** Diagnostic accuracy of heat exhaustion case definition using prehospital provider impressions as reference standard (n = 480).

Test characteristic	Value % [95% confidence interval]
Sensitivity	23.8 [12.1 – 39.5]
Specificity	93.6 [90.9 – 95.7]
Positive predictive value (2.1%, sample prevalence)	26.3 [13.4 – 43.1]
Negative predictive value (2.1%, sample prevalence)	92.8 [89.9 – 95.0]
Positive predictive value (11.9% prevalence)	33.5 [20.8 – 49.0]
Negative predictive value (11.9% prevalence)	90.1 [88.5 – 91.5]
Positive predictive value (20.1% prevalence)	48.4 [32.9 – 64.2]
Negative predictive value (20.1% prevalence)	83.0 [80.5 – 85.3]
True positives	10
False positives	28
True negatives	410
False negatives	32
Total positives, using prehospital provider impressions	42
Total positives, using case definitions	38

**Table 3:** Multivariate logistic regression analysis of risk factors associated with developing heat exhaustion, as determined by prehospital provider impressions (for n = 476 observations, with 4 observations dropped from the model due to missing exposure data).

Variable	Odds ratio	95% confidence interval	P-value
Station weather data			
Station heat index ≥ 49°C a	2.11	[0.54 – 8.22]	0.280
Station wind speed	0.97	[0.92 – 1.03]	0.368
Station visibility	0.69	[0.49 – 0.97]	0.034
On-scene environmental exposures			
Logger heat index ≥ 49°C^a^	2.66	[1.13 – 6.25]	0.025
On-scene air conditioning	0.29	[0.10 – 0.85]	0.024
Exposure to external heat source^b^	0.73	[0.26 – 2.08]	0.560
Behavioral history			
Recent history of exertion	3.66	[1.30 – 10.29]	0.014

a Re-coded as bivariate heat index thresholds;

b External heat sources, such as ovens.
