# Supplementary material for: Climate Change Adaptation: Prehospital Data Facilitate the Detection of Acute Heat Illness in India
Source: West J Emerg Med. 2021 Mar 24;22(3):739–49. doi: 10.5811/westjem.2020.11.48209 (PMC8203017; doi:10.5811/westjem.2020.11.48209)
Supplement: Supplementary file 1 [file wjem-22-739-s001.pdf]

Supplementary File for: "Pre-hospital data facilitate detection of acute heat illness in India: new tools for an emerging challenge"

Methods Supplement: Standardized questionnaire used by pre-hospital providers

Initial Patient Contact

Record ID ______________________

Call Info and Patient Demographics

Case ID Number ______________________

Chief complaint
- ◯ Abdominal pain
- ◯ BP Problem
- ◯ Breathing problem
- ◯ Cardiac problem
- ◯ Chest pain
- ◯ Diabetic problem
- ◯ Drunkenness
- ◯ Fainting
- ◯ Headache
- ◯ Mental problem
- ◯ Not breathing
- ◯ Seizures/convulsions
- ◯ Stroke
- ◯ Unconcious
- ◯ Fevers
- ◯ Vomiting

(These are from the GVK EMRI list of chief complaints. All ambulance dispatches with these chief complaints are to be screened for inclusion.)

Call Date and Time ______________________
(Date and time of 108 call.)

Activation Date and Time ______________________
(Date and time of ambulance dispatch.)

Information Source
- ◯ Patient
- ◯ Relative
- ◯ Bystander
- ◯ None

(Who was the primary source of information about the patient?)

Patient First Name ______________________
(If unknown, type Unknown.)

Patient Last Name ______________________
(If unknown, type Unknown.)

Patient Age ______________________
(Patient's age in years. Estimate if age is unknown.)

Patient Sex
- ◯ Male
- ◯ Female
- ◯ Not assessed

(Is the patient male or female?)

Patient literate?

◯ No
◯ Yes
◯ Unknown
(Can the patient read and write?)

Highest educational level completed

◯ No education or education less than 5th class
◯ Primary school (5th)
◯ Secondary (10th)
◯ Intermediate (12th)
◯ Graduate degree or above
◯ Not obtained
(Enter the highest level completed. Education below 5th standard should be included under "No education.")

Patient Phone Number

____
(Patient's phone number, including district area code. If unknown, enter Unknown.)

House Number

____
(Enter patient's house number and Taluka/Mandal. If unknown, enter Unknown.)

Scene and Transport Info

Scene Arrival Date and Time

____
(Date and time the ambulance arrived on scene.)

Pick Up Location

◯ Patient residence
◯ Other residence
◯ Worksite
◯ School or college
◯ Farm
◯ Public space
◯ Other, outdoors
◯ Other, indoors
◯ Health care facility

Patient Present?

◯ Yes
◯ No
(At the time the ambulance arrived on the scene, was the patient present? Reasons a patient may not be present include (but are not limited to) hoax calls, wrong address, patient relocated by the time ambulance arrived.)

Was patient transported?

◯ Yes, to hospital
◯ Yes, but aborted by patient request
◯ No, treated at scene and released
◯ No, patient refused transport

Scene Departure Date and Time

____
(Date and time the ambulance left the scene.)

Hospital Arrival Date and Time

____
(Date and time ambulance arrived at hospital.)

Hospital Name

____

Clinical Info

Body Temperature

____
(Patient's initial body temperature.)

Temperature measurement location

◯ oral
◯ axillary
◯ temporal
◯ rectal
◯ not measured
(Body location used to measure the patient's initial body temperature.)

Pulse Rate

____
(Patient's initial heart rate in beats per minute.)

Respiratory Rate

____
(Patient's initial respiratory rate in breaths per minute.)

Systolic BP

____
(Patient's initial systolic blood pressure in mm Hg.)

Diastolic BP

____
(Patient's initial diastolic blood pressure in mm Hg.)

Oxygen Saturation

____
(Patient's initial oxygen saturation on room air.)

Blood Glucose Level

____
(Patient's initial blood glucose level.)

GCS - Eye

◯ 1-Does not open eyes
◯ 2-Opens eyes in response to painful stimuli
◯ 3-Opens eyes in response to voice
◯ 4-Opens eyes spontaneously
◯ Not assessed
(Patient's initial GCS score for eye opening.)

GCS - Verbal

◯ 1-Makes no sound
◯ 2-Incomprehensible sounds
◯ 3-Utters inappropriate words
◯ 4-Confused, disoriented
◯ 5-Oriented, converses normally
◯ Not assessed
(Patient's initial GCS score for verbal.)

GCS - Motor

◯ 1-Makes no movements
◯ 2-Extension to painful stimuli
◯ 3-Abnormal flexion to painful stimuli
◯ 4-Flexion/withdrawal to painful stimuli
◯ 5-Localizes painful stimuli
◯ 6-Obeys commands
◯ Not assessed
(Patient's initial GCS score or motor.)

GCS Calculated Total

____
(Total initial GCS score. Automatic calculation, no need to enter data here.)

Patient Injured?

◯ Yes
◯ No
(Is an injury a major component of this patient's presentation?)

Pregnant?

◯ Yes
◯ No
◯ Unknown
◯ Not assessed
(Is the patient pregnant?)

What type of injury does the patient have?

____
(What type of injury does the patient have?)

Patient Occupation

____
(What is the patient's occupation?)

Occupational Setting

◯ Indoors
◯ Outdoors
◯ Mixed
◯ Unknown
◯ Not assessed
(What type of setting does the patient work in?)

External Heat Exposure

◯ Yes
◯ No
◯ Unknown
◯ Not assessed
(Was the patient exposed to any external heat sources (like an oven or stove) before getting sick?)

Illness Setting

◯ Home
◯ Work
◯ School or college
◯ Public space, outdoors
◯ Public space, indoors
◯ Other
◯ Not assessed
(Where was the patient when he or she got sick?)

Illness Setting Cooling

◯ Yes, air conditioning
◯ No
◯ Unknown
◯ Not assessed
(Is there any cooling (air conditioning, swamp cooler, fan) in the location where the patient became ill?)

Illness Setting and Pickup Location

◯ Yes
◯ No
(Is the setting where the patient became ill the same as the pickup location?)

Heavy Labor

◯ Yes
◯ No
◯ Unknown
◯ Not assessed
(Was the patient doing heavy labor prior to becoming ill?)

Felt Hot

◯ Yes
◯ No
◯ Unknown
◯ Not assessed
(Did the patient feel unusually hot before becoming ill?)

Last Water

◯ Within the last hour
◯ 2-3 hours
◯ 4-6 hours
◯ More than 6 hours
◯ Unknown
◯ Not assessed
(When did the patient last have a drink of water or other liquids?)

Past Medical History

☐ High blood pressure
☐ Diabetes
☐ Kidney disease
☐ Liver disease
☐ Seizures
☐ Alcohol abuse or dependence
☐ Other
☐ Unknown
☐ Not assessed
(What medical problems does the patient have? Check all that apply.)

Other Past Medical

____
(What other medical problems does the patient have?)

Cause of Emergency Code

____
(What is the medic's initial impression of the cause of the emergency? Use code from GVK EMRI PCR form.)
