# Supplementary material for: Climate Change Adaptation: Prehospital Data Facilitate the Detection of Acute Heat Illness in India
Source: West J Emerg Med. 2021 Mar 24;22(3):739–49. doi: 10.5811/westjem.2020.11.48209 (PMC8203017; doi:10.5811/westjem.2020.11.48209)

Supplementary File for: "Pre-hospital data facilitate detection of acute heat illness in India: new tools for an emerging challenge"

Figure S2: STARD 2015 flow diagram for heat stroke case definition

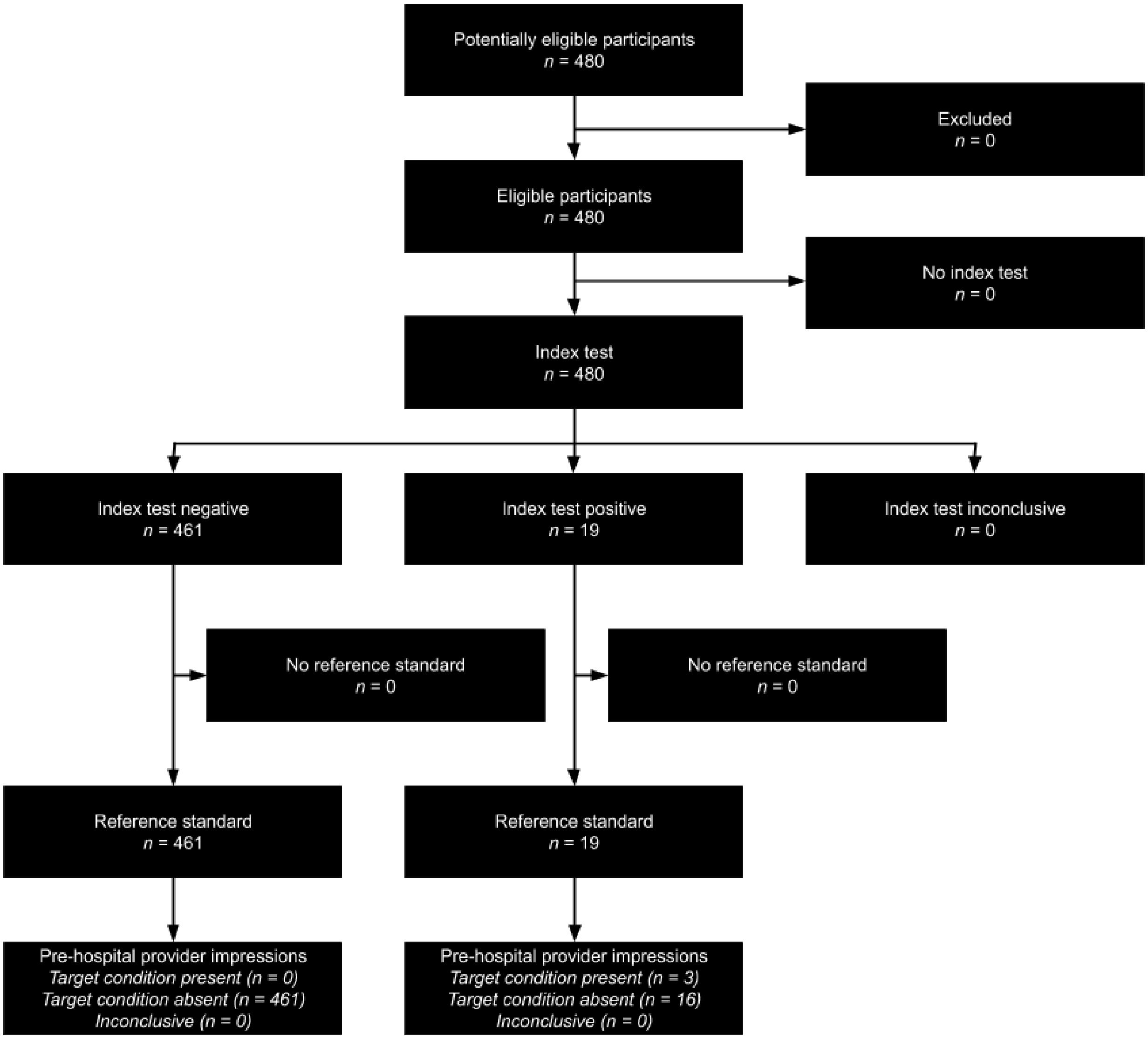

Supplement: Supplementary file 3 [file wjem-22-739-s003.pdf]
