# Supplementary material for: Climate Change Adaptation: Prehospital Data Facilitate the Detection of Acute Heat Illness in India
Source: West J Emerg Med. 2021 Mar 24;22(3):739–49. doi: 10.5811/westjem.2020.11.48209 (PMC8203017; doi:10.5811/westjem.2020.11.48209)
Supplement: Supplementary file 4 [file wjem-22-739-s004.docx]

**Supplementary File for: “Pre-hospital data facilitate detection of acute heat illness in India: new tools for an emerging challenge”**

| **Table S1.** T tests comparing mean heat indices calculated using data loggers and station data between heat exhaustion cases and non-cases as diagnosed by pre-hospital providers. | | | | | |
| --- | --- | --- | --- | --- | --- |
|  | *n*  Cases | *n*  Non-cases | Mean Heat Index  Cases | Mean Heat Index  Non-cases | *P* |
| **For Logger Heat Index** | 33 | 346 | 51.1 [49.7 - 52.5] | 49.9 [49.2 - 50.6] | 0.162 |
| In patients without history of heavy labor | 28 | 331 | 51.4 [49.9 - 52.9] | 50.0 [49.3 - 50.7] | 0.134 |
| In patients with history of heavy labor | 5 | 15 | 49.3 [43.0 - 55.7] | 48.4 [44.4 - 52.3] | 0.394 |
| **For Station Heat Index** | 42 | 438 | 44.6 [43.6 - 45.6] | 43.5 [43.2 - 43.9] | 0.037 |
| In patients without history of heavy labor | 36 | 418 | 44.6 [43.5 - 45.6] | 43.5 [43.1 - 43.9] | 0.050 |
| In patients with history of heavy labor | 6 | 20 | 44.8 [39.7 - 49.8] | 43.9 [42.1 - 45.7] | 0.327 |
