# Supplementary material for: Climate Change Adaptation: Prehospital Data Facilitate the Detection of Acute Heat Illness in India
Source: West J Emerg Med. 2021 Mar 24;22(3):739–49. doi: 10.5811/westjem.2020.11.48209 (PMC8203017; doi:10.5811/westjem.2020.11.48209)
Supplement: Supplementary file 5 [file wjem-22-739-s005.pdf]

STARD CHECKLIST

**TITLE OR ABSTRACT**	
**1**	**Identification as a study of diagnostic accuracy using at least one measure of accuracy (such as sensitivity, specificity, predictive values, or AUC)**
p.p. 1-2, "The case definition for heat exhaustion was 23.8% [12.1% - 39.5%] sensitive and 93.6% [90.9% - 95.7%] specific. The positive and negative predictive values were 33.5% [20.8% - 49.0%] and 90.1% [88.5% - 91.5%], respectively."	
**ABSTRACT**	
**2**	**Structured summary of study design, methods, results, and conclusions (for specific guidance, see STARD for Abstracts)**
p. 1, see introduction	
**INTRODUCTION**	
**3**	**Scientific and clinical background, including the intended use and clinical role of the index test**
p.p. 3-4, "We conducted an exploratory evaluation of three tools with the potential to facilitate early recognition of AHI and more accurate public health surveillance: (1) novel heat exhaustion and heat stroke case definitions for identifying likely AHI cases…" and "We evaluated the potential utility of these tools individually and in combination for improving diagnostic accuracy for AHI in the pre-hospital setting."	
**4**	**Study objectives and hypotheses**
**METHODS**	
Study Design	
**5**	**Whether data collection was planned before the index test and reference standard were performed (prospective study) or after (retrospective study)**
p. 4 "This study, which includes a retrospective and exploratory diagnostic accuracy evaluation and a cross-sectional analysis, was conducted between April - June 2016 in Ahmedabad, India. "<br><br>p. 8 "We conducted a retrospective and exploratory diagnostic accuracy evaluation of case definitions for heat exhaustion and heat stroke using pre-hospital provider clinical impressions and the initiation of cooling in the pre-hospital setting as a reference standard."	
Participants	
**6**	**Eligibility criteria**
p. 5 "We used a convenience sample of patients who were included if they presented to the GVK EMRI-operated "108" ambulance service from 8 AM to 8 PM with an eligible chief complaint during the study period and were served by one of six ambulance duty stations with the highest historical call volume for AHI. A list of eligible chief complaints consistent with AHI was developed a priori by the study team and included chest pain, abdominal pain, shortness of breath, intoxication, hypertension, hyper- or hypoglycemia, syncope, dysrhythmia, headache, mental health concerns, seizures, stroke, altered mental status, fever, and nausea and vomiting. All trauma activations were excluded from the study. Patients were enrolled from April 15 2016 - June 15 2016."	
**7**	**On what basis potentially eligible participants were identified (such as symptoms, results from previous tests, inclusion in registry)**
p. 5, as above.	
**8**	**Where and when potentially eligible participants were identified (setting, location, and dates)**
p. 5, as above.	
**9**	**Whether participants formed a consecutive, random, or convenience series**

p. 5, as above.	
Test Methods	
**10a**	**Index test, in sufficient detail to allow replication**
p. 8 "The heat exhaustion case definition included feeling hot with a complaint of any of the following: nausea, vomiting, dizziness, weakness, diarrhea, fainting, muscle cramps, hot and dry skin, hot and diaphoretic skin, or headache. The heat stroke case definition included a core temperature of at least 38.5 °C, with altered mental status (GCS less than or equal to 14, disorientation, seizures, or loss of consciousness). All temperatures obtained in the axilla were adjusted upward by 1 °C to more accurately reflect internal (i.e., rectal) temperatures."	
**10b**	**Reference standard, in sufficient detail to allow replication**
p. 8 "We conducted a retrospective and exploratory diagnostic accuracy evaluation of case definitions for heat exhaustion and heat stroke using pre-hospital provider clinical impressions and the initiation of cooling in the pre-hospital setting as a reference standard."	
**11**	**Rationale for choosing the reference standard (if alternatives exist)**
N/A (there were no alternative diagnoses available for this study).	
**12a**	**Definition of and rationale for test positivity cut-offs or result categories of the index test, distinguishing pre-specified from exploratory**
p. 8, as explained in items 10a and 10b above.	
**12b**	**Definition of and rationale for test positivity cut-offs or result categories of the reference standard, distinguishing pre-specified from exploratory**
N/A (reference standard was based on clinical impressions and did not include cut offs using, for example, laboratory markers).	
**13a**	**Whether clinical information and reference standard results were available to the performers or readers of the index test**
p. 8 "Regarding selection bias and uncertainty, index text results (i.e., the case definitions) were not available to pre-hospital providers in the field, however the study authors were not blinded to pre-hospital provider impressions while retroactively developing the case definitions."	
**13b**	**Whether clinical information and index test results were available to the assessors of the reference standard**
p. 8, as above	
Analysis	
**14**	**Methods for estimating or comparing measures of diagnostic accuracy**
p. 14 "The sensitivity, specificity, positive predictive values (PPV), and negative predictive values (NPV) of the heat exhaustion case definitions using pre-hospital provider impressions as the reference standard are shown in Table 2."	
**15**	**How indeterminate index test or reference standard results were handled**
N/A (index tests were based on exploratory analysis with no indeterminate results)	
**16**	**How missing data on the index test and reference standard were handled**
p. 11 "Due to technological malfunctioning of the data loggers, logger temperature and relative humidity were collected for 415 and 379 of the 480 participants, respectively, and missing values were dropped from the analysis."	
**17**	**Any analyses of variability in diagnostic accuracy, distinguishing pre-specified from exploratory**
See Table 2, and variability in PPV/NPV estimates based on assumed background prevalence.	

p. 9 "We also conducted a sensitivity analysis using multiple prevalence estimates to calculate positive and negative predictive values. Prevalence estimates were obtained from a previously conducted prevalence study of self-reported heat related symptoms (20.1%) and HRI (11.9%) among slum dwellers in Ahmedabad."	
**18**	**Intended sample size and how it was determined**
p.p. 8-9 "While we did not conduct sample size calculations, we anticipated a sample size of 300-600 participants based off of historical EMS call activity in the area during the summer months."	
**RESULTS**	
Participants	
**19**	**Flow of participants, using a diagram**
See Figures S1 and S2 (Supplementary material for heat exhaustion and heat stroke case definitions)	
**20**	**Baseline demographic and clinical characteristics of participants**
See Table 1	
**21a**	**Distribution of severity of disease in those with the target condition**
N/A (We did not capture disease severity with pre-hospital clinical data).	
**21b**	**Distribution of alternative diagnoses in those without the target condition**
N/A (We did not have access to final diagnoses)	
**22**	**Time interval and any clinical interventions between index test and reference standard**
N/A (This was retrospective and exploratory, clinical interventions were not conducted using index test results)	
**23**	**Cross tabulation of the index test results (or their distribution) by the results of the reference standard**
See Table 2	
**24**	**Estimates of diagnostic accuracy and their precision (such as 95% confidence intervals)**
See Table 2	
**25**	**Any adverse events from performing the index test or the reference standard**
N/A (This study did not involve invasive testing; it was based on history & physical data as well as clinical impressions).	
**DISCUSSION**	
**26**	**Study limitations, including sources of potential bias, statistical uncertainty, and generalisability**
p. 24, "Our study had several limitations. First, we relied on convenience sampling and a relatively small sample size. This likely underestimated the variability in on-scene temperature and the AHI predictive value of risk factors in the logistic regression analysis. Second, we relied on a passive data collection process and were not able to adequately capture and troubleshoot technological malfunctioning of the data loggers, which resulted in missing on-scene temperature and relative humidity data. Third, we were not able to obtain physician confirmed diagnoses and relied on pre-hospital provider impressions for our reference standards. However, pre-hospital providers in this area have been well trained in diagnosis and management of AHI following our prior efforts to develop a heat action plan in Ahmedabad 28. Last, there may be an element of selection bias: when diagnosing patients with AHI, pre-hospital providers may have been influenced by their of perception of heat at the location of patient pick-up."	
**27**	**Implications for practice, including the intended use and clinical role of the index test**
See Conclusion section, p. 24	
**OTHER INFORMATION**	

28	**Registration number and name of registry**
p. 10 "Ethics approval was obtained from the Indian Council of Medical Research (TRC/IEC No. 14/2015), the University of Washington (#51167), and the Indian Institute of Public Health, Gandhinagar."	
**29**	**Where the full study protocol can be accessed**
Data will be uploaded into an open access repository following acceptance.	
**30**	**Sources of funding and other support; role of funders**
p. 11 "This work was funded by the National Institutes of Health (grant number 5R21TW009535-02). The funding source had no involvement in study design, data collection or analysis, manuscript writing, or the decision to submit this manuscript for publication."	
