# Supplementary material for: Climate Change Adaptation: Prehospital Data Facilitate the Detection of Acute Heat Illness in India
Source: West J Emerg Med. 2021 Mar 24;22(3):739–49. doi: 10.5811/westjem.2020.11.48209 (PMC8203017; doi:10.5811/westjem.2020.11.48209)
Supplement: Supplementary file 6 [file wjem-22-739-s006.pdf]

STROBE Statement—Checklist of items that should be included in reports of ***cross-sectional studies***

	Item No	Recommendation
**Title and abstract**	1	(*a*) Indicate the study's design with a commonly used term in the title or the abstract<br><br>p. 1, Introduction: "In this exploratory cross-sectional and diagnostic accuracy study …"
		(*b*) Provide in the abstract an informative and balanced summary of what was done and what was found<br><br>p.p. 1-2 (see Methods and Results)
Introduction		
Background/rationale	2	Explain the scientific background and rationale for the investigation being reported<br><br>p. 3 "There is an unmet need to design, test, and evaluate tools to facilitate the early recognition and treatment of AHI in the pre-hospital setting and to facilitate public health surveillance. This deficit is particularly relevant in India, China, and other low- and middle-income countries (LMIC), which are disproportionately impacted by climate change and where extreme heat poses substantial risk 16–19."
Objectives	3	State specific objectives, including any prespecified hypotheses<br><br>p.p. 3-4 Paragraph 3 of introduction, "We conducted an exploratory evaluation of three tools with the potential to facilitate early recognition of AHI and more accurate public health surveillance: (1) novel heat exhaustion and heat stroke case definitions for identifying likely AHI cases; (2) portable digital temperature and humidity data loggers that can be used to measure real-time on-scene temperature at the time of first responder arrival; and (3) prevalence data for AHI risk factors contained in pre-hospital provider history and physicals (H&P) that can be used to facilitate early diagnosis."
Methods		
Study design	4	Present key elements of study design early in the paper

		p. 4 "This study, which includes a retrospective and exploratory diagnostic accuracy evaluation and a cross-sectional analysis, was conducted between April - June 2016 in Ahmedabad, India. "<br><br>See Figure 1
Setting	5	Describe the setting, locations, and relevant dates, including periods of recruitment, exposure, follow-up, and data collection<br><br>p. 4 "Ahmedabad is the sixth largest city in India. It has a population of 7.2 million people and is among the fastest growing cities in the country. It is also one of India's hottest cities, with summer maximum daily temperatures (T_{max}) averaging 45 °C from March – May. Like other industrializing cities in LMICs, several populations in Ahmedabad have a high risk for heat illness, including residents of slums and densely populated areas; individuals with limited access to water and air conditioning; and laborers in a range of settings.<br><br>p. 5 "A flow chart of study enrollment and data collection is shown in Figure 1. We used a convenience sample of patients who were included if they presented to the GVK EMRI-operated "108" ambulance service from 8 AM to 8 PM with an eligible chief complaint during the study period and were served by one of six ambulance duty stations with the highest historical call volume for AHI. A list of eligible chief complaints consistent with AHI was developed a priori by the study team and included chest pain, abdominal pain, shortness of breath, intoxication, hypertension, hyper- or hypoglycemia, syncope, dysrhythmia, headache, mental health concerns, seizures, stroke, altered mental status, fever, and nausea and vomiting. All trauma activations were excluded from the study. Patients were enrolled from April 15 2016 - June 15 2016."
Participants	6	(*a*) Give the eligibility criteria, and the sources and methods of selection of participants<br><br>p. 5 as above
Variables	7	Clearly define all outcomes, exposures, predictors, potential confounders, and effect modifiers. Give diagnostic criteria, if applicable<br><br>p. 9 "Unpaired t-tests were used to compare the difference in the mean heat index between AHI cases and non-cases, as diagnosed by pre-hospital providers, and we conducted sub-group analysis for participants with a reported history of recently experienced exertion, which may confound heat-health relationships. To compare logger and station heat indices among AHI cases, we conducted paired t-

		tests and used both pre-hospital provider impressions and the case definitions to reduce measurement uncertainty.”
Data sources/ measurement	8*	For each variable of interest, give sources of data and details of methods of assessment (measurement). Describe comparability of assessment methods if there is more than one group<br><br>See Figure 1
Bias	9	Describe any efforts to address potential sources of bias<br><br>p. 8 “Regarding selection bias and uncertainty, index text results (i.e., the case definitions) were not available to pre-hospital providers in the field, however the study authors were not blinded to pre-hospital provider impressions while retroactively developing the case definitions.”
Study size	10	Explain how the study size was arrived at<br><br>p.p. 8-9 “While we did not conduct sample size calculations, we anticipated a sample size of 300-600 participants based off of historical EMS call activity in the area during the summer months.”
Quantitative variables	11	Explain how quantitative variables were handled in the analyses. If applicable, describe which groupings were chosen and why<br><br>p. 9 “the mean heat index between AHI cases and non-cases”<br><br>p.p. 9-10 “models using each case definition as a dichotomous outcome variable” …. “Logger and station heat indices were included as dichotomous covariates”
Statistical methods	12	(*a*) Describe all statistical methods, including those used to control for confounding<br><br>p. 9 “We compared the heat index as measured by the data loggers with temperature measurements from the airport station and evaluated the correlation using Spearman’s rho.”… “Unpaired t-tests were used to compare the difference in the mean heat index between AHI cases and non-cases… “we conducted paired t-tests and used both pre-hospital provider impressions and the case definitions to reduce measurement uncertainty.”<br><br>p.p. 9-10 “A number of logistic regression models using each case definition as a dichotomous outcome variable (i.e., heat exhaustion and heat stroke cases and non-cases) were conducted to better characterize heat-health relationships, account for confounding variables…”

		(*b*) Describe any methods used to examine subgroups and interactions<br><br>p. 9 “and we conducted sub-group analysis for participants with a reported history of recently experienced exertion, which may confound heat-health relationships.”
		(*c*) Explain how missing data were addressed<br><br>p. 11 “Due to technological malfunctioning of the data loggers, logger temperature and relative humidity were collected for 415 and 379 of the 480 participants, respectively, and missing values were dropped from the analysis.”
		(*d*) If applicable, describe analytical methods taking account of sampling strategy<br><br>N/A
		(*e*) Describe any sensitivity analyses<br><br>p. 9 “We also conducted a sensitivity analysis using multiple prevalence estimates to calculate positive and negative predictive values. Prevalence estimates were obtained from a previously conducted prevalence study of self-reported heat related symptoms (20.1%) and HRI (11.9%) among slum dwellers in Ahmedabad.”
Results		
Participants	13*	(a) Report numbers of individuals at each stage of study—eg numbers potentially eligible, examined for eligibility, confirmed eligible, included in the study, completing follow-up, and analysed<br><br>See Figures S1 and S2.
		(b) Give reasons for non-participation at each stage<br><br>p. 11 “Due to technological malfunctioning of the data loggers, logger temperature and relative humidity were collected for 415 and 379 of the 480 participants, respectively, and missing values were dropped from the analysis.”
		(c) Consider use of a flow diagram<br><br>See Figures S1 and S2.

Descriptive data	14*	(a) Give characteristics of study participants (eg demographic, clinical, social) and information on exposures and potential confounders<br><br>See Table 1
		(b) Indicate number of participants with missing data for each variable of interest<br>See Table 1 as above
Outcome data	15*	Report numbers of outcome events or summary measures<br><br>See Figure 3, Figure 4, and Table 3. See p. 18 and Table S1.
Main results	16	(*a*) Give unadjusted estimates and, if applicable, confounder-adjusted estimates and their precision (eg, 95% confidence interval). Make clear which confounders were adjusted for and why they were included<br><br>See p. 18 and Table S1.
		(*b*) Report category boundaries when continuous variables were categorized<br><br>p. 10 “Given the clinical utility of thresholds, logger and station heat indices were included as dichotomous covariates with temperature thresholds ≥ or < 49°C (consistent with previously described heat wave temperature thresholds in South Asia 34), rather than as a continuous variable.”
		(*c*) If relevant, consider translating estimates of relative risk into absolute risk for a meaningful time period<br><br>N/A
Other analyses	17	Report other analyses done—eg analyses of subgroups and interactions, and sensitivity analyses<br><br>See Tables 2 (sensitivity analysis by disease prevalence) and Table S1 (subgroup analysis by activity history)
Discussion		
Key results	18	Summarise key results with reference to study objectives<br><br>p. 22 “We found a significant disparity between paired logger (on-scene) and station (METAR) heat indices, with scene temperatures being systematically warmer.”

		p. 22 “This is reinforced by the finding that heat exhaustion cases were significantly and positively associated with a logger heat index ≥ 49 °C, indicating that scene temperature and relative humidity may have utility as environmental tests for AHI in the pre-hospital setting.”<br><br>p. 22 “a history of exertion seems to have substantially lowered the temperature threshold for developing AHI. We also found that access to air conditioning was negatively associated with a diagnosis of heat exhaustion.”<br><br>p. 23 “Our case definition for heat exhaustion had a low sensitivity of 23.8% and PPV of 26.3%, assuming a background prevalence of 11.9 %”
Limitations	19	Discuss limitations of the study, taking into account sources of potential bias or imprecision. Discuss both direction and magnitude of any potential bias<br><br>p. 24 “Our study had several limitations. First, we relied on convenience sampling and a relatively small sample size. This likely underestimated the variability in on-scene temperature and the AHI predictive value of risk factors in the logistic regression analysis. Second, we relied on a passive data collection process and were not able to adequately capture and troubleshoot technological malfunctioning of the data loggers, which resulted in missing on-scene temperature and relative humidity data. Third, we were not able to obtain physician confirmed diagnoses and relied on pre-hospital provider impressions for our reference standards. However, pre-hospital providers in this area have been well trained in diagnosis and management of AHI following our prior efforts to develop a heat action plan in Ahmedabad. Last, there may be an element of selection bias: when diagnosing patients with AHI, pre-hospital providers may have been influenced by their of perception of heat at the location of patient pick-up.”
Interpretation	20	Give a cautious overall interpretation of results considering objectives, limitations, multiplicity of analyses, results from similar studies, and other relevant evidence<br><br>p. 24 “Despite these limitations, the findings from our study clearly suggest that adding additional data to pre-hospital evaluations for AHI can improve diagnostic accuracy, even in a setting with an ambulance service that is highly attuned to AHI. In particular, scene temperature, a history of exertion prior to illness onset, and presence of air conditioning are valuable data points. Collecting data on scene temperature is feasible and improves exposure estimation,

		particularly for patients with AHI. Having a standing strategy for collecting additional data regarding activity and scene environment are likely important for early AHI detection."
Generalisability	21	Discuss the generalisability (external validity) of the study results<br><br>p. 25 "These practice modifications can facilitate adaptation to climate change, which is increasing the frequency and severity of extreme heat events. Our findings may have particular relevance to other cities in LMICs with centrally-administered EMS systems and environmental conditions similar to Ahmedabad's."
Other information		
Funding	22	Give the source of funding and the role of the funders for the present study and, if applicable, for the original study on which the present article is based<br><br>p. 11 "This work was funded by the National Institutes of Health (grant number 5R21TW009535-02). The funding source had no involvement in study design, data collection or analysis, manuscript writing, or the decision to submit this manuscript for publication."

*Give information separately for exposed and unexposed groups.
